# Extrasynaptic Neurotransmission Mediated by Exocytosis and Diffusive Release of Transmitter Substances

**DOI:** 10.3389/fnsyn.2018.00013

**Published:** 2018-06-08

**Authors:** Elaine Del-Bel, Francisco F. De-Miguel

**Affiliations:** ^1^Department of Morphology Physiology and Basic Pathology, Dental School of Ribeirão Preto, USP-Center for Interdisciplinary Research on Applied Neurosciences (NAPNA), University of São Paulo (USP), São Paulo, Brazil; ^2^Instituto de Fisiología Celular-Neurociencias, Centro de Ciencias de la Complejidad, Universidad Nacional Autónoma de México, Mexico City, Mexico

**Keywords:** transmitter release, exocytosis, extrasynaptic transmission, volume transmission, diffusive transmitters

## Abstract

This review article deals with the mechanisms of extrasynaptic release of transmitter substances, namely the release from the soma, axon and dendrites in the absence of postsynaptic counterparts. Extrasynaptic release occurs by exocytosis or diffusion. Spillover from the synaptic cleft also contributes to extrasynaptic neurotransmission. Here, we first describe two well-known examples of exocytosis from the neuronal soma, which may release copious amounts of transmitter for up to hundreds of seconds after electrical stimulation. The mechanisms for somatic exocytosis of the low molecular weight transmitter serotonin, and the peptides oxytocin and vasopressin have been studied in detail. Serotonin release from leech neurons and oxytocin and vasopressin from rodent neurons have a common multi-step mechanism, which is completely different from that for exocytosis from presynaptic endings. Most transmitters and peptides released extrasynaptically seem to follow this same mechanism. Extrasynaptic exocytosis may occur onto glial cells, which act as intermediaries for long-term and long-distance transmission. The second part of this review article focuses on the release upon synthesis of the representative diffusible molecules nitric oxide (NO) and endocannabinoids. Diffusible molecules are synthesized “on demand” from postsynaptic terminals in response to electrical activity and intracellular calcium elevations. Their effects include the retrograde modulation of presynaptic electrical activity and transmitter release. Extrasynaptic neurotransmission is well exemplified in the retina. Light-evoked extrasynaptic communication sets the gain for visual responses and integrates the activity of neurons, glia and blood vessels. Understanding how extrasynaptic communication changes the function of hard-wired circuits has become fundamental to understand the function of the nervous system.

## Introduction

The demonstration by Santiago Ramon y Cajal of the existence of stereotyped circuits in the nervous system, followed by the discovery that acetylcholine, adrenaline and noradrenaline are released by nerve terminals by Elliot (1904), Loewi (1921) and Dale et al. (1936) set the basis for the discoveries by Bernard Katz and his colleagues on the fundamental mechanism for synaptic communication (for review see Katz, 1996). The later discovery of electrical junctions (Furshpan and Potter, 1959) complemented the dominating concept that neuronal circuits function in a hard-wired manner. However, with time it also became clear that the input/output relationship of neuronal circuits varies depending on the previous patterns of electrical activity. Short- and long-term synaptic plasticity explains of some transitory changes in the strength of the circuit connectivity. However, extrasynaptic communication explains the integral modulation of whole neuronal circuits, glia and blood vessels in periods ranging from seconds to hours.

The classical observations of Dalstrom and Fuxe that the serotonin cell bodies in the Raphe nucleus are surrounded by free serotonin (reviewed by Fuxe et al., 2007; Borroto-Escuela et al., 2015), and the observation by Paton and Vizi (1969) that biogenic amines inhibit non-synaptically the cholinergic transmission onto muscle fibers suggested that transmitters may act extrasynaptically. The evidence was soon expanded to other transmitters and peptides, thus leading to the mechanistic concept of volume transmission, defined by Fuxe et al. (2007) as a form of communication mediated by extracellular diffusion of transmitter substances through the extracellular space (for review see Borroto-Escuela et al., 2015). Indirect evidence for the somatic release of transmitters came from experiments by Dun and Minota (1982) showing that electrical stimulation of the soma of peripheral neurons changed the membrane potential in a non-synaptic manner. Direct demonstrations of the extrasynaptic release of all sorts of low molecular transmitters and peptides came later, from experiments in central and peripheral neurons of vertebrates and invertebrates (for review see Trueta and De-Miguel, 2012). This bulk of evidence lead to the term of “extrasynaptic communication” to define volume transmission in response to transmitter liberation from extrasynaptic sites, in the soma, dendrites and axons (De-Miguel and Nicholls, 2015). Extrasynaptic release occurs in the absence of postsynaptic counterparts. In addition, synaptically-released transmitters, for example, dopamine, noradrenaline or glutamate (Zhang and Sulzer, 2003; Rice and Cragg, 2008; Courtney and Ford, 2014) spillover from the synaptic cleft and reach extrasynaptic receptors, thus contributing to extrasynaptic communication within small volumes of tissue. Glial cells are integral components of extrasynaptic communication by responding to transmitters and peptides and releasing the same or others. As will be seen below, the capillary blood flow is a target for extrasynaptic modulation.

Extrasynaptic release of transmitters occurs in central and peripheral neurons of vertebrates and invertebrates. Most low molecular weight transmitters and different peptides are released extrasynaptically (Trueta and De-Miguel, 2012). In addition, gases such as nitric oxide (NO), carbon monoxide (Queiroga et al., 2015) and hydrogen sulfide (Paul and Snyder, 2018), or the liposoluble endocannabinoid family (Iannotti et al., 2016; Lu and Mackie, 2016) and the hydrogen peroxide (Lee et al., 2015), are synthesized “*on demand,”* and reach their targets retrogradely (usually presynaptic), by diffusion.

In this mini-review article, we have assumed the immense task of comparing the mechanisms of release by exocytosis and by the synthesis of diffusible molecules. To achieve this goal, we first compare the best-known release mechanisms of classic transmitters, peptides, NO and cannabinoids. Then, we take advantage of the well-known retinal structure and function to give an account on how synaptic and extrasynaptic transmission interact to modulate visual sensitivity and blood flow.

## The Mechanism for Extrasynaptic Exocytosis

**Figure 1:**
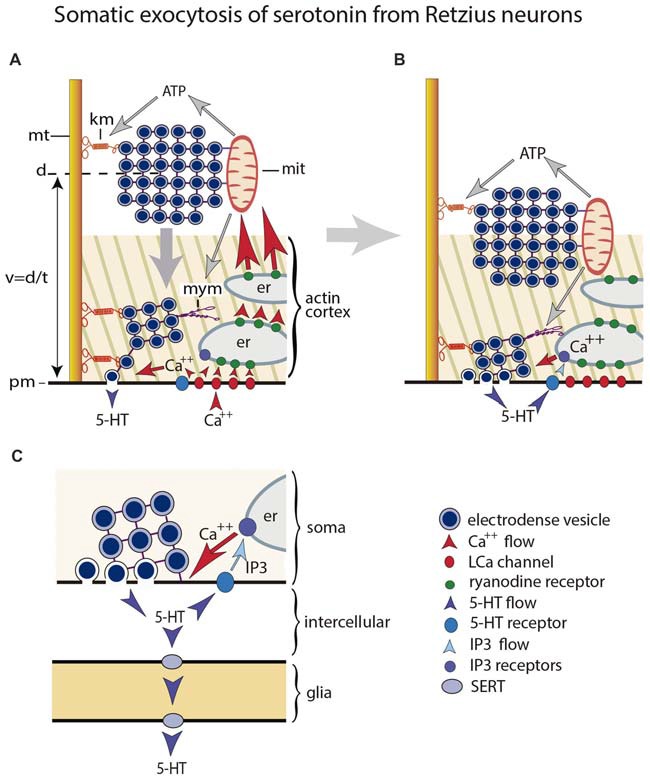
Mechanism for somatic exocytosis of serotonin in leech Retzius neuron. **(A)** Vesicles forming clusters rest at different distances from the plasma membrane. Clusters are attached to microtubules via kinesin motors. At rest the actin cortex restricts vesicle mobilization. Clusters already inside the cortex spontaneously send vesicles to the plasma membrane where they release serotonin. Trains of electrical impulses promote calcium entry through L channels. Calcium induces calcium release from endoplasmic reticulum via ryanodine receptor activation. The amplified calcium wave arrives at the mitochondria, which responds producing ATP. The kinesin myosin motors become activated by ATP, thus transporting the vesicle clusters towards the plasma membrane. Electrical activity and calcium change the configuration of the actin cortex, which now becomes permeable for vesicle transport, with incorporation of myosin motors. The vesicle clusters are propelled towards the plasma. **(B)** Arrival of vesicle clusters at the plasma membrane occurs seconds after electrical activity and the intracellular calcium wave ended. The large-scale exocytosis is produced by a positive feedback loop established by serotonin released by the individual vesicles. The serotonin that has been released activates autoreceptors and phospholipase C. IP3 activates calcium release from the external layer of endoplasmic reticulum. This calcium maintains exocytosis until the last vesicles in the cluster fuse. **(C)** Amplified scheme of the positive feedback system, introducing the glia as serotonin transporter. Hypothetically this transport occurs via SER transporters that introduce serotonin when the internal concentration is low and release it at distal sites when the internal concentration is high.

This section compares the extrasynaptic exocytosis of the low molecular weight transmitter serotonin and the peptides oxytocin and vasopressin. The release mechanism of both substances has been studied step by step in great detail. Somatic exocytosis of serotonin, resumed schematically in Figure 1, has been studied in the large soma of the classical Retzius neuron of the leech (De-Miguel et al., 2015); somato-dendritic oxytocin and vasopressin have been studied in thalamic mammalian neurons (Ludwig and Leng, 2006; Ludwig and Stern, 2015). The accessibility of both neuron types has permitted to apply diverse technical approaches in the search for direct experimental evidence on the exocytosis mechanism. The mechanism for both types of molecules are remarkable similar, and quite different from that for synaptic exocytosis. Since one example comes from release of a low molecular weight transmitter in an invertebrate and the other from peptides in mammals, the similarity predicts universal mechanistic steps governing somatic exocytosis. The cumulative evidence obtained from other central and peripheral neuron types from vertebrates and invertebrates, releasing low molecular transmitters or peptides strengthen this hypothesis (for review see Trueta and De-Miguel, 2012).

Serotonin, oxytocin and vasopressin are packed in large (~100 nm) electrodense vesicles (a definition that stems from their appearance under the electron microscope) that rest at a distance from the plasma membrane (Coggeshall, 1972; Schimchowitsch et al., 1983). Single action potentials fail to evoke the large-scale exocytosis that characterizes these somata. However, rapid trains of impulses or large depolarizations, trigger a massive exocytosis that lasts for hundreds of seconds (Trueta et al., 2003; Ludwig and Stern, 2015). The frequencies of the trains of impulses that produce somatic exocytosis of serotonin are physiological, and can be evoked by mechanosensory stimulation to the skin (Velázquez-Ulloa et al., 2003).

The coupling between excitation and exocytosis incorporates a cascade of sequential steps. The large intracellular calcium transient produced mostly by its entry through L channels (Trueta et al., 2004; Tobin et al., 2011) activates an intracellular calcium-induced calcium release that generates a calcium “tsunami” that invades the whole soma (Sabatier et al., 1997; Ludwig et al., 2002; Tobin et al., 2011; Leon-Pinzon et al., 2014). As result, vesicles become transported actively to the plasma membrane across an actin cortex (Tobin and Ludwig, 2007; Tobin et al., 2011; De-Miguel et al., 2012). In serotonergic neurons, vesicle clusters are carried by molecular motors over 0.6–6.0 mm distances at 15–90 nm/s velocities (De-Miguel et al., 2012). This transport determines the characteristic long latency of the large-scale somatic exocytosis, which starts seconds after the end of the calcium transient produced by electrical stimulation (Leon-Pinzon et al., 2014). Somatic exocytosis is maintained by a transmitter and calcium positive feedback loop (Wotjak et al., 1994; Leon-Pinzon et al., 2014). In serotonergic neurons, activation of 5-HT2 receptors by the serotonin that has been released increases the calcium concentration in the soma shell via the activation of phospholipase C and the production of IP3. This calcium elevation, promotes exocytosis as new vesicles arrive, but its localization prevents the transport of vesicle clusters resting more internally (Leon-Pinzon et al., 2014). The feedback loop ends when the last vesicles in the pool fuse with the plasma membrane. Strikingly enough, somatic exocytosis of serotonin in leech neurons occurs onto glial cells (Trueta et al., 2004). As will be seen below, in peripheral neurons and in retina, activation of glial cells by extrasynaptic exocytosis extends the duration and consequences of extrasynaptic communication.

Evidence from mammalian serotonergic Raphe neurons points to a similar mechanism for somatic exocytosis. The capacity of serotonin to emit fluorescence upon multiphoton excitation allowed the group of Sudipta Maiti (Kaushalya et al., 2008; Sarkar et al., 2012) to study somatic exocytosis in Raphe neurons isolated from rodents. Depolarization with a high potassium extracellular solution triggers the mobilization of fluorescent serotonergic-containing spots to the plasma membrane followed by exocytosis. The size of these fluorescence spots resembles that of vesicle clusters in Retzius neurons.

Most neurotransmitters are released extrasynaptically and follow the mechanistic rules described above for serotonin and peptides (Trueta and De-Miguel, 2012). A well-studied example is dopamine release from amacrine cells in the retina of rodents and from invertebrate neurons (Chen et al., 1996; Puopolo et al., 2001). The somata and dendrites of dopaminergic neurons in the substantia nigra and the ventral tegmental area also release dopamine (Björklund and Lindvall, 1975; Geffen et al., 1976; Cheramy et al., 1981). However, there are also certain variations: somatic release of dopamine in basal ganglia seems to occur from clear vesicles (Jaffe et al., 1998). The functional effects of extrasynaptic release of dopamine in the retina are discussed below.

## Extrasynaptic Transmission Mediated by the Synthesis of Diffusible Molecules: The Nitrergic and Endocannabinoid Systems

A separate set of diffusible transmitters is synthesized on demand upon increases of electrical activity and activation of certain G protein-coupled receptors, both of which produce increases of intracellular calcium concentration. Most diffusible neurotransmitters are liberated upon one or two rapid enzymatic steps after which they diffuse to the extracellular space. Therefore there is no defined mechanism for their release. Gases like NO, carbon monoxide and hydrogen sulfate diffuse freely across plasma membranes. They activate specific cellular and molecular targets (Bredt and Snyder, 1990; Wang, 2002). Another family of diffusible transmitters, the endocannabinoids, are the endogenous activators of the specific receptors that respond to chemicals produced by the plant cannabis. Endocannabinoids are also produced on demand by a receptor-stimulated cleavage of membrane phospholipid precursors. Once synthesized, endocannabinoids also diffuse rapidly out the neurons (Piomelli, 2003; Mechoulam and Parker, 2013).

## The Nitric Oxide (NO) System

Because of its physicochemical nature, NO is a volume transmitter (Agnati et al., 2010; Garthwaite, 2016). Increases in the intracellular calcium levels in the presence of the enzyme nitric-oxide-synthase (NOS), a calcium-calmodulin enzyme, produces NO from its precursor L-arginine (see Garthwaite et al., 1988; Bredt and Snyder, 1990; Garthwaite, 2008, 2016). The mechanism of NO inactivation remains unclear, although cytochrome P450 oxidoreductase and astrocytes seem to contribute (Hall et al., 2009; Rodriguez-Grande and Konsman, 2018).

The best-characterized stimulator of NO synthesis is the massive exocytosis of glutamate from hippocampal presynaptic endings (Garthwaite, 2008, 2016). During low presynaptic activity, glutamate activates mostly postsynaptic AMPA/kainate receptors. However, an increased glutamate release upon bursts of presynaptic action potentials promotes the opening of NMDA receptors. The pore of NMDA receptors is highly permeable to calcium that concentrates in the postsynaptic terminal. NMDA receptors and nNOS are associated by the postsynaptic density protein-95 (Brenman et al., 1996; Sattler et al., 1999). In this way, calcium influx activates the NOS/calmodulin complex that produces NO. The NO synthesis continues as long as the calcium levels are elevated. The NO synthesis is also activated by the cytoplasmic calcium increases in response to the activation of voltage-gated calcium channels or intracellular calcium release (Daniel et al., 1998).

Right after being synthesized, NO diffuses through aqueous and lipid environments, thus acting on pre- and postsynaptic targets. Unlike transmitters released by exocytosis, NO lacks enough chemical sophistication to activate specific receptor binding sites. However, it may activate the soluble enzyme guanylyl cyclase that converts guanosine-5′-triphosphate into cyclic guanosine-monophosphate (Arnold et al., 1977). NO also produces the nitrosylation of proteins and the generation of reactive oxygen species (Ahern et al., 2002).

The NO targets are presynaptic terminals, glia and blood vessels. For this reason NO is a retrograde transmitter. The physicochemical properties of NO allow its uniform diffusion bypassing most if not all anatomical constrains. NO may act in concert with other transmitters, producing subtle alterations in the function of ion channels and other proteins (for review see Steinert et al., 2010). Moreover, NO may link monoaminergic and glutamatergic transmission (West and Grace, 2000; Kiss and Vizi, 2001; Mitkovski et al., 2012). An excess of NO synthesis becomes neurotoxic by the formation of reactive oxygen species.

NO produces in rats and schizophrenic patients a rapid and long-lasting improvement of anxiety and depressive symptoms (Guimarães et al., 1994; Issy et al., 2011; Hallak et al., 2013). The inhibition of NO synthesis produces anxiolytic-, antidepressant-, anti-fear and anti-traumatic like effects (for review see Guimarães et al., 2005; Steinert et al., 2010; Paul and Snyder, 2018). In addition, reduces the L-DOPA-induced that follows the depletion of dopaminergic neurons in rodents and non-human primates (Del Bel et al., 2005; Padovan-Neto et al., 2009; Bortolanza et al., 2015; for review see Del-Bel et al., 2011). For these reason, all of these symptoms seem to have extrasynaptic communication components and open a field of study from the view of extrasynaptic communication.

## The Endocannabinoid System

Endocannabinoids also contribute to extrasynaptic communication. The production of cannabinoids also occurs on demand in postsynaptic endings (Kano et al., 2009; Castillo et al., 2012; Iannotti et al., 2016; Lu and Mackie, 2016), upon increases of electrical activity (Piomelli, 2003; Mechoulam and Parker, 2013). Constitutive membrane phospholipids like di-and tri-acylglycerols are metabolized intracellularly by a calcium-dependent diacylglycerol-lipase, to produce the best-known cannabinoids: anandamide and 2-arachidonoylglycerol (2-AG; for review, see Bisogno et al., 2005). Anandamide is synthesized upon activation of phospholipase β-coupled- glutamate, muscarinic or dopamine receptors. The synthesis of 2-AG requires an intracellular calcium elevation, as it happens for NO. The increases in the calcium concentration activate the enzyme N-arachidonoyl-phosphatidyl ethanolamine-specific phospholipase. Anandamide and 2-AG leak passively by diffusing throughout lipid membranes. However, a rapid and selective carrier accelerates this process in neurons and glial cells (Beltramo et al., 1997). Endocannabinoids are also secreted in extracellular membrane vesicles originated in microglial cells (Gabrielli et al., 2015).

Endocannabinoids are retrograde modulators of synaptic function by acting on specific presynaptic and glial cannabinoid receptors (reviewed in Hashimotodani et al., 2007; Kano et al., 2009). The type 1 (CB1) and type 2 (CB2) specific cannabinoid receptors belong to the G protein-coupled receptor family. A third cannabinoid receptor is the transient receptor potential vanilloid type 1 (TRPV1). Endocannabinoids may also modulate synapses by using glial cells as intermediaries. In addition, glial cells also produce endocannabinoids (Stella, 2010).

Termination of the endocannabinoid signaling occurs through a carrier-mediated transport into cells, followed by intracellular degradation (Piomelli, 2003; Iannotti et al., 2016; Lu and Mackie, 2016). 2AG is mostly degraded in presynapse by the enzyme monoacylglycerol lipase (Dinh et al., 2002; Marrs et al., 2010), while anandamide is degraded mostly in postsynapses by the fatty acid amide hydrolase to produce arachidonic acid and ethanolamine (Di Marzo et al., 1994; for review, see Iannotti et al., 2016; Lu and Mackie, 2016).

When we fell down as children, our grandmothers massaged our hurt knees to reduce our pain. A good explanation for grandmother’s empirical knowledge came from experiments made in leech. Endocannabinoids released upon stimulation of touch- or pressure-mechanosensory neurons innervating the skin, act on TRPV receptors to decrease nociceptive synaptic transmission and increase the responses of the touch and pressure sensory connections (Summers et al., 2017).

Endocannabinoids modulate the excitatory and inhibitory synaptic strength of sensorymotor pathways (Pedrazzi et al., 2015). However, that endocannabinoid receptors appear more prominently in inhibitory terminals suggest their function to reduce over-excitability (Freund et al., 2003; Chevaleyre et al., 2006; for review, see Iannotti et al., 2016; Lu and Mackie, 2016). Endocanncabinoids inhibit transmitter release by closing calcium channels, opening K^+^ channels, inhibiting adenylyl cyclase and stimulating protein kinases (Kano et al., 2009; Castillo et al., 2012). In addition, activation of CB1 receptors increase the spontaneous firing of noradrenergic, serotonergic and dopaminergic neurons, and increases the synthesis of these neurotransmitters (Mechoulam and Parker, 2013). Endocannabinoids also improve certain regeneration processes (Kwiatkoski et al., 2012) and increase neurogenesis (Campos et al., 2016). The endocannabinoid system may also be neuroprotector and a target to control neurodegenerative and neuropsychiatric diseases (for review see Campos et al., 2012, 2016).

## Integration of Synaptic and Extrasynaptic Transmission in the Retina

In this section, we will use the excellent possibilities offered by the histological organization and supercomputing power of the retina to exemplify how extrasynaptic communication integrates the function of neurons, glia and blood vessels. The link between extrasynaptic communication and its effects in the retina has been widely studied for dopamine, with some examples in the contribution of NO and cannabinoids. However, these substances suffice to exemplify the wide spectrum of concerted extrasynaptic communication actions that modulate function in a well-known neural tissue.

A bright light shone onto a retinal receptive field evokes electric signals in photoreceptors. On their way to the ganglion cells, interactions with bipolar, horizontal and amacrine cells, produce the characteristic “on” and “off” visual responses. In addition, activation of amacrine cells evoke the extrasynaptic exocytosis of dopamine (Puopolo et al., 2001) and GABA (Hirasawa et al., 2009). The mechanism for dopamine release (Puopolo et al., 2001) is as described for serotonin and oxytocin. Through volume transmission, dopamine increases the gain of the sensory field by three complementary effects: (a) potentiating the activity of glutamate receptors in bipolar and horizontal cells (Knapp and Dowling, 1987; Maguire and Werblin, 1994); (b) reducing the diameter of the visual field by uncoupling horizontal cells (Piccolino et al., 1984; DeVries and Schwartz, 1989); and (c) uncoupling the connections of AII rod amacrine cells and modifying the center-surround balance in ganglion cells (Daw et al., 1990).

Increases in the extracellular concentration of transmitters, activates the retinal glia—the Muller cells. The Muller cells respond by releasing ATP through a special type of channels, the pannexins (Dahl, 2015). ATP depresses the electrical activity of ganglion cells and evokes vasodilation of blood vessels (Newman, 2015).

Diffusible transmitters also contribute to retinal function. The blockade of NO synthesis increases blood pressure (Deussen et al., 1993). In addition, the activation of cannabinoid receptors reduces L calcium and K currents in cones while increases L currents and reduces K currents in rhodes (Straiker et al., 1999).

## Discussion and Perspectives

Extrasynaptic transmission is multivariate in every region of the nervous system. Several modes and sites of transmitter release exist different neurons. In addition, one neuron can be modulated by different transmitters. All modes of extrasynaptic release are triggered by increases of electrical activity, followed by large increases in the intracellular calcium concentration. Signaling is slow when compared to synaptic communication, which occurs within half a millisecond. The threshold and amount of each extrasynaptic mode of release are coded by the frequency and duration of the stream of action potentials. In return, extrasynaptically released transmitters modulate, and in most cases reduce, the neuronal electrical activity.

Axons and dendrites contain clusters of clear and dense core vesicles anchored at different distances of the plasma membrane. These diverse configurations endow neurons with regional release possibilities. Passage of electrical activity along the neuron may then trigger different modes of exocytosis. Depending on the transmitter released and the region where release occurs, extrasynaptic exocytosis may have different timing and regional effects.

The ample catalog of transmitter molecules and extrasynaptic receptors contributing to extrasynaptic signaling adds a wide range of activity-dependent physiological responses to neuronal circuits. This contributes to explain the diversity of circuit responses, depending on the activity levels. Extrasynaptic communication incorporates glia, which adds feedback communication to neurons, releases chemical messages and regulates blood flow. Therefore, to understand the function of the nervous system, it is now essential to understand the roles of extrasynaptic neurotransmission.

Several pertinent questions that can be addressed now concern how many release modes a single neuron has? How release from different neuronal compartments modulates activity locally? How extrasynaptic release produces a self-modulation? How can we relate extrasynaptic neurotransmission to motivation, modulation, state-dependence or activity-dependence? Although not touched for the case of extrasynaptic exocytosis, an important question that can now be posted is the contribution of extrasynaptic communication in normal and diseased brain. Several examples were discussed for diffusible transmitters. For some diseases like depression or Parkinson’s, the demonstrations of the role of extrasynaptic exocytosis of low molecular transmitters and peptides seems around the corner.

## Author Contributions

ED-B and FD-M contributed equally to each step of the preparation of this manuscript.

## Conflict of Interest Statement

The authors declare that the research was conducted in the absence of any commercial or financial relationships that could be construed as a potential conflict of interest.

## References

[B107] Agnati L. F. Guidolin D. Guescini M. Genedani S. Fuxe K. (2010). Understanding wiring and volume transmission. Brain Res. Rev. 64, 137–159. 10.1016/j.brainresrev.2010.03.003 20347870

[B1] Ahern G. P. Klyachko V. A. Jackson M. B. (2002). cGMP and S-nitrosylation: two routes for modulation of neuronal excitability by NO. Trends Neurosci. 25, 510–517. 10.1016/s0166-2236(02)02254-3 12220879

[B2] Arnold W. P. Mittal C. K. Katsuki S. Murad F. (1977). Nitric oxide activates guanylate cyclase and increases guanosine 3′:5′-cyclic monophosphate levels in various tissue preparations. Proc. Natl. Acad. Sci. U S A 74, 3203–3207. 10.1073/pnas.74.8.3203 20623 PMC431498

[B3] Beltramo M. Stella N. Calignano A. Lin S. Y. Makriyannis A. Piomelli D. (1997). Functional role of high-affinity anandamide transport, as revealed by selective inhibition. Science 277, 1094–1097. 10.1126/science.277.5329.1094 9262477

[B4] Bisogno T. Ligresti A. Di Marzo V. (2005). The endocannabinoid signalling system: biochemical aspects. Pharmacol. Biochem. Behav. 81, 224–238. 10.1016/j.pbb.2005.01.027 15935454

[B5] Björklund A. Lindvall O. (1975). Dopamine in dendrites of substantia nigra neurons: suggestions for a role in dendritic terminals. Brain Res. 83, 531–537. 10.1016/0006-8993(75)90849-5 1111820

[B6] Borroto-Escuela D. O. Agnati L. F. Bechter K. Jansson A. Tarakanov A. O. Fuxe K. (2015). The role of transmitter diffusion and flow versus extracellular vesicles in volume transmission in the brain neural-glial networks. Philos. Trans. R. Soc. Lond. B Biol. Sci. 370:20140183. 10.1098/rstb.2014.0183 26009762 PMC4455752

[B7] Bortolanza M. Padovan-Neto F. E. Cavalcanti-Kiwiatkoski R. Dos Santos-Pereira M. Mitkovski M. Raisman-Vozari R.. (2015). Are cyclooxygenase-2 and nitric oxide involved in the dyskinesia of Parkinson’s disease induced by L-DOPA? Philos. Trans. R. Soc. Lond. B Biol. Sci. 370:20140190. 10.1098/rstb.2014.0190 26009769 PMC4455759

[B8] Bredt D. S. Snyder S. H. (1990). Isolation of nitric oxide synthetase, a calmodulin-requiring enzyme. Proc. Natl. Acad. Sci. U S A 87, 682–685. 10.1073/pnas.87.2.682 1689048 PMC53329

[B9] Brenman J. E. Chao D. S. Gee S. H. McGee A. W. Craven S. E. Santillano D. R.. (1996). Interaction of nitric oxide synthase with the postsynaptic density protein PSD-95 and α1-syntrophin mediated by PDZ domains. Cell 84, 757–767. 10.1016/s0092-8674(00)81053-3 8625413

[B10] Campos A. C. Fogaça M. V. Sonego A. B. Guimarães F. S. (2016). Cannabidiol, neuroprotection and neuropsychiatric disorders. Pharmacol. Res. 112, 119–127. 10.1016/j.phrs.2016.01.033 26845349

[B11] Campos A. C. Moreira F. A. Gomes F. V. Del Bel E. A. Guimarães F. S. (2012). Multiple mechanisms involved in the large-spectrum therapeutic potential of cannabidiol in psychiatric disorders. Philos. Trans. R. Soc. Lond. B Biol. Sci. 367, 3364–3378. 10.1098/rstb.2011.0389 23108553 PMC3481531

[B12] Castillo P. E. Younts T. J. Chávez A. E. Hashimotodani Y. (2012). Endocannabinoid signaling and synaptic function. Neuron 76, 70–81. 10.1016/j.neuron.2012.09.020 23040807 PMC3517813

[B13] Chen G. Gutman D. A. Zerby S. E. Ewing A. G. (1996). Electrochemical monitoring of bursting exocytotic events from the giant dopamine neuron of *Planorbis corneus*. Brain Res. 733, 119–124. 10.1016/s0006-8993(96)00754-8 8891256

[B14] Cheramy A. Leviel V. Glowinski J. (1981). Dendritic release of dopamine in the substantia nigra. Nature 289, 537–543. 10.1038/289537a0 6258083

[B15] Chevaleyre V. Takahashi K. A. Castillo P. E. (2006). Endocannabinoid-mediated synaptic plasticity in the CNS. Annu. Rev. Neurosci. 29, 37–76. 10.1146/annurev.neuro.29.051605.112834 16776579

[B16] Coggeshall R. E. (1972). Autoradiographic and chemical localization of 5-hydroxytryptamine in identified neurons in the leech. Anat. Rec. 172, 489–498. 10.1002/ar.1091720303 4536825

[B17] Courtney N. A. Ford C. P. (2014). The timing of dopamine- and noradrenaline-mediated transmission reflects underlying differences in the extent of spillover and pooling. J. Neurosci. 34, 7645–7656. 10.1523/JNEUROSCI.0166-14.2014 24872568 PMC4035525

[B18] Dahl G. (2015). ATP release through pannexon channels. Philos. Trans. R. Soc. Lond. B Biol. Sci. 370:20140191. 10.1098/rstb.2014.0191 26009770 PMC4455760

[B19] Dale H. H. Feldberg W. Vogt M. (1936). Release of acetylcholine at voluntary motor nerve endings. J. Physiol. 86, 353–380. 10.1113/jphysiol.1936.sp003371 16994763 PMC1394683

[B20] Daniel H. Levenes C. Crépel F. (1998). Cellular mechanisms of cerebellar LTD. Trends Neurosci. 21, 401–407. 10.1016/s0166-2236(98)01304-6 9735948

[B21] Daw N. W. Jensen R. J. Brunken W. J. (1990). Rod pathways in mammalian retinae. Trends Neurosci. 13, 110–115. 10.1016/0166-2236(90)90187-f 1691871

[B22] Del Bel E. A. Guimarães F. S. Bermúdez-Echeverry M. Gomes M. Z. Schiaveto-de-souza A. Padovan-Neto F. E.. (2005). Role of nitric oxide on motor behavior. Cell. Mol. Neurobiol. 25, 371–392. 10.1007/s10571-005-3065-8 16047547 PMC11529539

[B23] Del-Bel E. Padovan-Neto F. E. Raisman-Vozari R. Lazzarini M. (2011). Role of nitric oxide in motor control: implications for Parkinson’s disease pathophysiology and treatment. Curr. Pharm. Des. 17, 471–488. 10.2174/138161211795164176 21375483

[B24] De-Miguel F. F. Leon-Pinzon C. Noguez P. Mendez B. (2015). Serotonin release from the neuronal cell body and its long-lasting effects on the nervous system. Philos. Trans. R. Soc. Lond. B Biol. Sci. 370:20140196. 10.1098/rstb.2014.0196 26009775 PMC4455765

[B25] De-Miguel F. F. Nicholls J. G. (2015). Release of chemical transmitters from cell bodies and dendrites of nerve cells. Philos. Trans. R. Soc. Lond. B Biol. Sci. 370:20140181. 10.1098/rstb.2014.0181 26009760 PMC4455750

[B26] De-Miguel F. F. Santamaría-Holek I. Noguez P. Bustos C. Hernández-Lemus E. Rubí J. M. (2012). Biophysics of active vesicle transport, an intermediate step that couples excitation and exocytosis of serotonin in the neuronal soma. PLoS One 7:e45454. 10.1371/journal.pone.0045454 23056204 PMC3463611

[B27] Deussen A. Sonntag M. Vogel R. (1993). L-arginine-derived nitric oxide: a major determinant of uveal blood flow. Exp. Eye Res. 57, 129–134. 10.1006/exer.1993.1107 8405178

[B28] DeVries S. H. Schwartz E. A. (1989). Modulation of an electrical synapse between solitary pairs of catfish horizontal cells by dopamine and second messengers. J. Physiol. 414, 351–375. 10.1113/jphysiol.1989.sp017692 2558170 PMC1189146

[B29] Di Marzo V. Fontana A. Cadas H. Schinelli S. Cimino G. Schwartz J. C.. (1994). Formation and inactivation of endogenous cannabinoid anandamide in central neurons. Nature 372, 686–691. 10.1038/372686a0 7990962

[B30] Dinh T. P. Carpenter D. Leslie F. M. Freund T. F. Katona I. Sensi S. L.. (2002). Brain monoglyceride lipase participating in endocannabinoid inactivation. Proc. Natl. Acad. Sci. U S A 99, 10819–10824. 10.1073/pnas.152334899 12136125 PMC125056

[B31] Dun N. J. Minota S. (1982). Post-tetanic depolarization in sympathetic neurones of the guinea-pig. J. Physiol. 323, 325–337. 10.1113/jphysiol.1982.sp014075 7097577 PMC1250359

[B32] Elliot T. R. (1904). On the action of adrenalin. J. Physiol. 31, 20–21.

[B33] Freund T. F. Katona I. Piomelli D. (2003). Role of endogenous cannabinoids in synaptic signaling. Physiol. Rev. 83, 1017–1066. 10.1152/physrev.00004.2003 12843414

[B34] Furshpan E. J. Potter D. D. (1959). Transmission at the giant motor synapses of the crayfish. J. Physiol. 145, 289–325. 10.1113/jphysiol.1959.sp006143 13642302 PMC1356828

[B35] Fuxe K. Dahlström A. Höistad M. Marcellino D. Jansson A. Rivera A.. (2007). From the Golgi-Cajal mapping to the transmitter-based characterization of the neuronal networks leading to two modes of brain communication: wiring and volume transmission. Brain Res. Rev. 55, 17–54. 10.1016/j.brainresrev.2007.02.009 17433836

[B36] Gabrielli M. Battista N. Riganti L. Prada I. Antonucci F. Cantone L.. (2015). Active endocannabinoids are secreted on extracellular membrane vesicles. EMBO Rep. 16, 213–220. 10.15252/embr.201439668 25568329 PMC4328748

[B37] Garthwaite J. (2008). Concepts of neural nitric oxide-mediated transmission. Eur. J. Neurosci. 27, 2783–2802. 10.1111/j.1460-9568.2008.06285.x 18588525 PMC2610389

[B39] Garthwaite J. (2016). From synaptically localized to volume transmission by nitric oxide. J. Physiol. 594, 9–18. 10.1113/JP270297 26486504 PMC4704503

[B38] Garthwaite J. Charles S. L. Chess-Williams R. (1988). Endothelium-derived relaxing factor release on activation of NMDA receptors suggests role as intercellular messenger in the brain. Nature 336, 385–388. 10.1038/336385a0 2904125

[B40] Geffen L. B. Jessell T. M. Cuello A. C. Iversen L. L. (1976). Release of dopamine from dendrites in rat substantia nigra. Nature 260, 258–260. 10.1038/260258a0 1256567

[B41] Guimarães F. S. Beijamini V. Moreira F. A. Aguiar D. C. de Lucca A. C. (2005). Role of nitric oxide in brain regions related to defensive reactions. Neurosci. Biobehav. Rev. 29, 1313–1322. 10.1016/j.neubiorev.2005.03.026 16095696

[B42] Guimarães F. S. de Aguiar J. C. Del Bel E. A. Ballejo G. (1994). Anxiolytic effect of nitric oxide synthase inhibitors microinjected into the dorsal central grey. Neuroreport 5, 1929–1932. 10.1097/00001756-199410000-00022 7531004

[B43] Hallak J. E. Maia-de-Oliveira J. P. Abrao J. Evora P. R. Zuardi A. W. Crippa J. A.. (2013). Rapid improvement of acute schizophrenia symptoms after intravenous sodium nitroprusside: a randomized, double-blind, placebo-controlled trial. JAMA Psychiatry 70, 668–676. 10.1001/jamapsychiatry.2013.1292 23699763

[B108] Hall C. N. Keynes R. G. Garthwaite J. (2009). Cytochrome P450 oxidoreductase participates in nitric oxide consumption by rat brain. Biochem. J. 419, 411–418. 10.1042/BJ20082419 19152507 PMC2662488

[B44] Hashimotodani Y. Ohno-Shosaku T. Kano M. (2007). Presynaptic monoacylglycerol lipase activity determines basal endocannabinoid tone and terminates retrograde endocannabinoid signaling in the hippocampus. J. Neurosci. 27, 1211–1219. 10.1523/JNEUROSCI.4159-06.2007 17267577 PMC6673197

[B45] Hirasawa H. Puopolo M. Raviola E. (2009). Extrasynaptic release of GABA by retinal dopaminergic neurons. J. Neurophysiol. 102, 146–158. 10.1152/jn.00130.2009 19403749 PMC2712262

[B102] Iannotti F. A. Di Marzo V. Petrosino S. (2016). Endocannabinoids and endocannabinoid-related mediators: targets, metabolism and role in neurological disorders. Prog. Lipid Res. 62, 107–128. 10.1016/j.plipres.2016.02.002 26965148

[B46] Issy A. C. Lazzarini M. Szawka R. E. Carolino R. O. Anselmo-Franci J. A. Del Bel E. A. (2011). Nitric oxide synthase inhibitors improve prepulse inhibition responses of Wistar rats. Behav. Brain Res. 217, 416–423. 10.1016/j.bbr.2010.11.016 21074571

[B47] Jaffe E. H. Marty A. Schulte A. Chow R. H. (1998). Extrasynaptic vesicular transmitter release from the somata of substantia nigra neurons in rat mid brain slices. J. Neurosci. 18, 3548–3553. 10.1523/JNEUROSCI.18-10-03548.1998 9570786 PMC6793140

[B48] Kano M. Ohno-Shosaku T. Hashimotodani Y. Uchigashima M. Watanabe M. (2009). Endocannabinoid-mediated control of synaptic transmission. Physiol. Rev. 89, 309–380. 10.1152/physrev.00019.2008 19126760

[B49] Katz B. (1996). Neural transmitter release: from quantal secretion to exocytosis and beyond. The Fenn Lecture. J. Neurocytol. 12, 677–686. 10.1007/bf02284834 9023717

[B50] Kaushalya S. K. Desai R. Arumugam S. Ghosh H. Balaji J. Maiti S. (2008). Three-photon microscopy shows that somatic release can be a quantitatively significant component of serotonergic neurotransmission in the mammalian brain. J. Neurosci. Res. 86, 3469–3480. 10.1002/jnr.21794 18709651

[B51] Kiss J. P. Vizi E. S. (2001). Nitric oxide: a novel link between synaptic and nonsynaptic transmission. Trends Neurosci. 24, 211–2115. 10.1016/s0166-2236(00)01745-8 11250004

[B52] Knapp A. G. Dowling J. E. (1987). Dopamine enhances excitatory amino-acid gated conductances in cultured retinal horizontal cells. Nature 325, 437–439. 10.1038/325437a0 2880299

[B53] Kwiatkoski M. Guimarães F. S. Del-Bel E. (2012). Cannabidiol-treated rats exhibited higher motor score after cryogenic spinal cord injury. Neurotox. Res. 21, 271–280. 10.1007/s12640-011-9273-8 21915768

[B104] Lee C. R. Patel J. C. O’Neill B. Rice M. E. (2015). Inhibitory and excitatory neuromodulation by hydrogen peroxide: translating energetics to information. J. Physiol. 593, 3431–3446. 10.1113/jphysiol.2014.273839 25605547 PMC4560576

[B54] Leon-Pinzon C. Cercós M. G. Noguez P. Trueta C. De-Miguel F. F. (2014). Exocytosis of serotonin from the neuronal soma is sustained by a serotonin and calcium-dependent feedback loop. Front. Cell. Neurosci. 8:169. 10.3389/fncel.2014.00169 25018697 PMC4072984

[B56] Loewi O. (1921). Uber humorale ubertragbarkeit der herznernvirkung. I. Mittelung. Pflugers Arch. 189, 239–242.

[B57] Ludwig M. Leng G. (2006). Dendritic peptide release and peptide-dependent behaviours. Nat. Rev. Neurosci. 7, 126–136. 10.1038/nrn1845 16429122

[B58] Ludwig M. Sabatier N. Bull P. M. Landgraf R. Dayanithi G. Leng G. (2002). Intracellular calcium stores regulate activity-dependent neuropeptide release from dendrites. Nature 418, 85–89. 10.1038/nature00822 12097911

[B59] Ludwig M. Stern J. (2015). Multiple signalling modalities mediated by dendritic exocytosis of oxytocin and vasopressin. Philos. Trans. R. Soc. Lond. B Biol. Sci. 370:20140182. 10.1098/rstb.2014.0182 26009761 PMC4455751

[B103] Lu H. C. Mackie K. (2016). An introduction to the endogenous cannabinoid system. Biol. Psychiatry 79, 516–525. 10.1016/j.biopsych.2015.07.028 26698193 PMC4789136

[B60] Maguire G. Werblin F. (1994). Dopamine enhances a glutamate-gated ionic current in OFF bipolar cells of the tiger salamander retina. J. Neurosci. 14, 6094–6101. 10.1523/JNEUROSCI.14-10-06094.1994 7931565 PMC6576970

[B61] Marrs W. R. Blankman J. L. Horne E. A. Thomazeau A. Lin Y. H. Coy J.. (2010). The serine hydrolase ABHD6 controls the accumulation and efficacy of 2-AG at cannabinoid receptors. Nat. Neurosci. 13, 951–957. 10.1038/nn.2601 20657592 PMC2970523

[B62] Mechoulam R. Parker L. A. (2013). The endocannabinoid system and the brain. Annu. Rev. Psychol. 64, 21–47. 10.1146/annurev-psych-113011-143739 22804774

[B63] Mitkovski M. Padovan-Neto F. E. Raisman-Vozari R. Ginestet L. da-Silva C. A. del-Bel E. A. (2012). Investigations into potential extrasynaptic communication between the dopaminergic and nitrergic systems. Front. Physiol. 3:372. 10.3389/fphys.2012.00372 23055978 PMC3457048

[B64] Newman E. A. (2015). Glial cell regulation of neuronal activity and blood flow in the retina by release of gliotransmitters. Philos. Trans. R. Soc. Lond. B Biol. Sci. 370:20140195. 10.1098/rstb.2014.0195 26009774 PMC4455764

[B65] Padovan-Neto F. E. Echeverry M. B. Tumas V. Del-Bel E. A. (2009). Nitric oxide synthase inhibition attenuates L-DOPA-induced dyskinesias in a rodent model of Parkinson’s disease. Neuroscience 159, 927–935. 10.1016/j.neuroscience.2009.01.034 19302833

[B66] Paton W. D. M. Vizi E. S. (1969). The inhibitory action of noradrenaline and adrenaline on acetylcholine output by guinea-pig ileum longitudinal muscle strip. British J. Pharmacol. 35, 10–28. 10.1111/j.1476-5381.1969.tb07964.x 4302725 PMC1703074

[B101] Paul B. D. Snyder S. H. (2018). Gasotransmitter hydrogen sulfide signaling in neuronal health and disease. Biochem. Pharmacol. 149, 101–109. 10.1016/j.bcp.2017.11.019 29203369 PMC5868969

[B67] Pedrazzi J. F. Issy A. C. Gomes F. V. Guimarães F. S. Del-Bel E. A. (2015). Cannabidiol effects in the prepulse inhibition disruption induced by amphetamine. Psychopharmacology 232, 3057–3065. 10.1007/s00213-015-3945-7 25943166

[B68] Piccolino M. Neyton J. Gerschenfeld H. M. (1984). Decrease of gap junction permeability induced by dopamine and cyclic adenosine 3′:5′-monophosphate in horizontal cells of turtle retina. J. Neurosci. 4, 2477–2488. 10.1523/JNEUROSCI.04-10-02477.1984 6092564 PMC6564702

[B69] Piomelli D. (2003). The molecular logic of endocannabinoid signaling. Nat. Rev. Neurosci. 4, 873–884. 10.1038/nrn1247 14595399

[B70] Puopolo M. Hochstetler S. E. Gustincich S. Wightman R. M. Raviola E. (2001). Extrasynaptic release of dopamine in a retinal neuron: activity dependence and transmitter modulation. Neuron 30, 211–225. 10.1016/s0896-6273(01)00274-4 11343656

[B100] Queiroga C. S. Vercelli A. Vieira H. L. (2015). Carbon monoxide and the CNS: challenges and achievements. Br. J. Pharmacol. 172, 1533–1545. 10.1111/bph.12729 24758548 PMC4369262

[B71] Rice M. E. Cragg S. J. (2008). Dopamine spillover after quantal release: rethinking dopamine transmission in the nigrostriatal pathway. Brain Res. Rev. 58, 303–313. 10.1016/j.brainresrev.2008.02.004 18433875 PMC2879278

[B72] Rodriguez-Grande B. Konsman J. P. (2018). Gas diffusion in the CNS. J. Neurosci. Res. 96, 207–218. 10.1002/jnr.24077 28504343

[B73] Sabatier N. Richard P. Dayanithi G. (1997). L-, N- and T- but neither P- nor Q-type Ca^2+^ channels control vasopressin-induced Ca^2+^ influx in magnocellular vasopressin neurones isolated from the rat supraoptic nucleus. J. Physiol. 503, 253–268. 10.1111/j.1469-7793.1997.253bh.x 9306270 PMC1159860

[B74] Sarkar B. Das A. K. Arumugam S. Kaushalya S. K. Bandyopadhyay A. Balaji J.. (2012). The dynamics of somatic exocytosis in monoaminergic neurons. Front. Physiol. 3:414. 10.3389/fphys.2012.00414 23133421 PMC3490137

[B75] Sattler R. Xiong Z. Lu W. Y. Hafner M. MacDonald J. F. Tymianski M. (1999). Specific coupling of NMDA receptor activation to nitric oxide neurotoxicity by PSD-95 protein. Science 284, 1845–1848. 10.1126/science.284.5421.1845 10364559

[B76] Schimchowitsch S. Stoeckel M. E. Klein M. J. Garaud J. C. Schmitt G. Porte A. (1983). Oxytocin-immunoreactive nerve fibers in the pars intermedia of the pituitary in the rabbit and hare. Cell Tissue Res. 228, 255–263. 10.1007/bf00204877 6130846

[B77] Steinert J. R. Chernova T. Forsythe I. D. (2010). Nitric oxide signaling in brain function, dysfunction and dementia. Neuroscientist 16, 435–452. 10.1177/1073858410366481 20817920

[B78] Stella N. (2010). Cannabinoid and cannabinoid-like receptors in microglia, astrocytes, and astrocytomas. Glia 58, 1017–1030. 10.1002/glia.20983 20468046 PMC2919281

[B79] Straiker A. Stella N. Piomelli D. Mackie K. Karten H. J. Maguire G. (1999). Cannabinoid CB1 receptors and ligands in vertebrate retina: localization and function of an endogenous signaling system. Proc. Natl. Acad. Sci. U S A 96, 14565–14570. 10.1073/pnas.96.25.14565 10588745 PMC24476

[B80] Summers T. Hanten B. Peterson W. Burrell B. (2017). Endocannabinoids have opposing effects on behavioral responses to nociceptive and non-nociceptive stimuli. Sci. Rep. 7:5793. 10.1038/s41598-017-06114-1 28724917 PMC5517658

[B81] Tobin V. A. Douglas A. J. Leng G. Ludwig M. (2011). The involvement of voltage-operated calcium channels in somato-dendritic oxytocin release. PLoS One 6:e25366. 10.1371/journal.pone.0025366 22028774 PMC3197583

[B82] Tobin V. A. Ludwig M. (2007). The role of the actin cytoskeleton in oxytocin and vasopressin release from rat supraoptic nucleus neurons. J. Physiol. 582, 1337–1348. 10.1113/jphysiol.2007.132639 17478532 PMC2075266

[B83] Trueta C. De-Miguel F. F. (2012). Extrasynaptic exocytosis and its mechanisms: a source of molecules mediating volume transmission in the nervous system. Front. Physiol. 3:319. 10.3389/fphys.2012.00319 22969726 PMC3432928

[B85] Trueta C. Méndez B. De-Miguel F. F. (2003). Somatic exocytosis of serotonin mediated by L-type calcium channels in cultured leech neurones. J. Physiol. 547, 405–416. 10.1113/jphysiol.2002.030684 12562971 PMC2342656

[B86] Trueta C. Sánchez-Armass S. Morales M. A. De-Miguel F. F. (2004). Calcium-induced calcium release contributes to somatic secretion of serotonin in leech Retzius neurons. J. Neurobiol. 61, 309–316. 10.1002/neu.20055 15389693

[B105] Velázquez-Ulloa N. Blackshaw S. E. Szczupak L. Trueta C. García E. De-Miguel F. F. (2003). Convergence of mechanosensory inputs onto neuromodulatory serotonergic neurons in the leech. J. Neurobiol. 54, 604–617. 10.1002/neu.10184 12555272

[B87] Wang R. (2002). Two’s company, three’s a crowd: can H2S be the third endogenous gaseous transmitter? FASEB J. 16, 1792–1798. 10.1096/fj.02-0211hyp 12409322

[B88] West A. R. Grace A. A. (2000). Striatal nitric oxide signaling regulates the neuronal activity of midbrain dopamine neurons *in vivo*. J. Neurophysiol. 83, 1796–1808. 10.1152/jn.2000.83.4.1796 10758092

[B89] Wotjak C. T. Ludwig M. Landgraf R. (1994). Vasopressin facilitates its own release within the rat supraoptic nucleus *in vivo*. Neuroreport 5, 1181–1184. 10.1097/00001756-199406020-00005 7919160

[B91] Zhang H. Sulzer D. (2003). Glutamate spillover in the striatum depresses dopaminergic transmission by activating group I metabotropic glutamate receptors. J. Neurosci. 23, 10585–10592. 10.1523/JNEUROSCI.23-33-10585.2003 14627643 PMC6740919

